# No Effect of the Right Posterior Parietal Cortex tDCS in Dual-Target Visual Search

**DOI:** 10.3389/fpsyg.2018.02112

**Published:** 2018-11-12

**Authors:** Alyona A. Lanina, Matteo Feurra, Elena S. Gorbunova

**Affiliations:** ^1^Laboratory of Digital Interface User’s Cognitive Psychology, National Research University Higher School of Economics, Moscow, Russia; ^2^Centre for Cognition & Decision Making, Institute of Cognitive Neuroscience, National Research University Higher School of Economics, Moscow, Russia; ^3^Laboratory of Digital Interface User’s Cognitive Psychology, School of Psychology, National Research University Higher School of Economics, Moscow, Russia

**Keywords:** visual search, subsequent search misses, tDCS, posterior parietal cortex, visual attention

## Abstract

“Subsequent search misses” represent a decrease in accuracy at detecting a second target in a visual search task. In this study, we tested the possibility to modulate this effect via inhibition of the right posterior parietal cortex trough transcranial direct current stimulation (tDCS). The target stimuli were T-shapes presented among L-shaped distractors. The participant’s task was to detect targets or to report their absence. For each trial, targets could be represented by one high-salient target, one low-salient target, two different targets (one high salient and one low salient), two high salient targets, two low salient targets, or no targets at all (catch-trials). Offline tDCS was applied over the right (target site) or left (control site) posterior parietal cortex. Sham stimulation over the right posterior parietal cortex was included as a control (placebo). Stimulation lasted for 10 min. Afterward, participants were asked to perform the experiment. Our findings suggest that stimulation did not modulate any of the task conditions, suggesting potential limitation of the study: either tDCS was not enough powerful to modulate the task performance or the task was too easy to be modulated by stimulation.

## Introduction

Visual search for targets among distractors is a task that people face in everyday life. This search consists of finding both ordinary and important things intended to meet a particular need. An example is the search for banned substances or weapons in the hand luggage, which is conducted by the airport control personnel. Such type of search can be analyzed in terms of similarity of targets and distractors and through the conditions under which the visual search task is accomplished.

In most of the visual search tasks, only one target is required to be found. If it is found, then the search can be immediately stopped. If the target is not found then the search takes approximately twice as long (Adamo et al., 2013). These single-target situations are investigated more than multiple-target situations. Nevertheless, in the real world, there is often not only one target but several ones related to the same type or category. For example, it may be that a person who undergoes radiological screening has more than one tumor or a person’s hand baggage can be spotted by airport security check for more than one prohibited item. In everyday life, missing more than one target can lead to bad consequences. The type of error where the subsequent targets are skipped after the first target is detected, is widespread and originally known as the “satisfaction of search” [e.g., the “SOS” (Fleck et al., 2010; Tuddenham, 1962)]. This effect has been studied by researchers in the field of radiology for 50 years. Although the focus of research on SOS was given to radiologists using medical imaging, recent data have shown that the SOS occurs in basic visual search tasks as well (Fleck et al., 2010). Initially, it was thought that SOS occurs when the subject finishes the search prematurely, since they remain “satisfied” after finding the first target (Tuddenham, 1962). However, this position is not empirically justified (Berbaum, 2012). Alternative theories have been supposed to explain the fact that subjects generally do not exhibit SOS, but on the contrary, they often spend the same amount of time in searching regardless of whether one or more targets were present. In relation to that, the phenomenon of missing additional target after finding the first one, formerly referred to the SOS, was renamed to “Subsequent Search Misses” [SSM, (Adamo et al., 2013)].

Several studies, both in the field of radiology and in the field of cognitive psychology, have shown that a “similarity bias” can be a potential cause of the SSM in tasks that include the search for several targets (Biggs et al., 2015). In the study, Biggs et al. (2015) showed that the magnitude of the SSM effect is decreased when first and second targets are identical, compared to the condition that they are different. Also, the amplitude of the SSM effect was reduced when the second target was perceptually or categorically similar to the first found target. Moreover, later studies revealed the role of perceptual similarity (the presence of identical features in the first and the second target) – SSM amplitude decreased when the number of shared features in two targets increased (Gorbunova, 2017).

Another explanation for the “similarity bias” refers to the retention of a particular object in the working memory (WM). In most of the SSM studies, participants find the first target and press the appropriate key button. Such an explicit and conscious interaction with the first stimulus may lead to the creation of a representation of the stimulus with certain perceptive and categorical attributes inside the WM. The retention of these features in the WM can shift the attention to other stimuli that have perceptual or categorical features similar to the first stimulus. This idea is consistent with the finding of Cain et al. (2014), which revealed that several single-target search tasks effectively free the WM resources used by the first found target and reduce the amplitude of the SSM.

The relation between WM and attention can be described in terms of the biased competition model (Desimone and Duncan, 1995). According to this model, the objects in the visual field compete for cognitive processing. This competition is biased toward the objects that are currently attended in the visual field or most relevant to behavior, whereas the top-down bias is related mostly to working memory processing. The selection mechanism proposed by this model may be relevant to resource depletion account to SSM errors (Berbaum et al., 1991). In particular, perceptual and categorical attentional set can be the outcome of a depletion of available resources: after finding the first target, subjects can make the best use of their remaining cognitive resources to shift the attention toward the subsequent search.

Subsequent search misses effect bears a striking resemblance to the phenomenon observed in rapid serial visual presentation (RSVP) tasks – a sort of temporal search – known as “attentional blink” (Shapiro et al., 1994). In a standard “attentional blink” paradigm, stimuli are presented rapidly in the same place of the visual field and the subject’s task is to identify the two targets that are presented. The “attentional blink” phenomenon refers to a decrease in accuracy in the detection of the second target (T2), when it is presented from 200 to 500 ms after the first target (T1) is correctly identified. The resource-depletion theory which states that the locations and identities of the found targets are stored in the WM and consume cognitive resources that can be used for subsequent searches (Cain and Mitroff, 2013), is the potential explanation for the “attentional blink.” The resource-depletion theory may also be due to the SSM.

In the attempt to understand the potential common mechanism for the phenomena of SSM and “attentional blink,” Adamo et al. (2013) conducted an experiment using the eye-tracker to determine fixations on targets. The analysis of fixation lags (the number of fixations between T1 and T2) and temporal bins (the time between the offset from fixating T1 and the onset of fixating T2) was made. The study revealed that the decrease in the accuracy of finding the second target occurred by analogy with the position-dependent decrease in the “attentional blink” both for fixation-based and time-based analysis: accuracy at detecting T2 decreased at lag 2 and recovered at lag 4, as well as it decreased at 135–405 ms and recovered at 405–675 ms. Despite the differences between the paradigms used to identify the “attentional blink” and the SSM, a similar behavioral pattern was revealed, suggesting hypothetical common psychological and neural mechanisms for these phenomena. However, the link between the SSM and the attentional processing is not completely clear. Although there are obvious differences in the methods used to identify the typical effects of the “attentional blink” and the SSM (RSVP and visual search task), both methods are focused on the similar process - the omission of the second target after detecting or identifying the first one.

The effect of “SSM” is relatively recent, and it is far from being fully understood. Moreover, the neural mechanisms of attention are not clear too. The classical point of view on attention-related phenomena assumes the involvement of right parietal cortex (Wojciulik and Kanwisher, 1999). Cooper et al. (2004) study, the subjects conducted a standard “attentional blink” task in which they had to identify the first target (T1) and subsequently the second target (T2). During the experiment, subjects received repeated Transcranial Magnetic Stimulation (TMS) of the posterior site over the interhemispheric fissure (electrode site Pz, control site) and the right posterior parietal cortex (PPC, electrode site P4). Stimulation of the right PPC reduced the magnitude of “attentional blink,” while stimulation of the control site did not reveal any change.

Non Invasive Brain Stimulation (NIBS) techniques such as Transcranial Direct Current Stimulation (tDCS) and TMS have been successfully applied for investigation of perceptual and attentional processes. TMS is known to affect visual-spatial perception in landmark tasks (Ricci et al., 2012; Salatino et al., 2014b) and judgments about the symmetry of prebisected lines (Fierro et al., 2000). Additionally, it has been shown that TMS can simulate spatial neglect in healthy volunteers (Sack, 2010). tDCS is known to affect visual detection processes (Sparing et al., 2009), detection and discrimination of single and multiple competing stimuli (Filmer et al., 2015), performance in partial report task (Moos et al., 2012) and attentional reorienting processes (Roy et al., 2015). NIBS are used in the clinical practice such as to help people to recover from strokes (Salatino et al., 2014a; D’Agata et al., 2016). It should be noted that the main advantage of NIBS is the possibility to establish causal links between the behavior and the stimulated brain areas.

The action mechanism underlying tDCS is simple. Electrodes are applied to the head, through which a weak electrical current (1–2 mA) is delivered over the human scalp. The current strength is typically too low to induce neurons to produce action potential. However, the electrical current induces significant changes at the level of the neuronal physiological state (membrane action potential). These changes induce the cortical neurons to be more or less prone to activation, depending on the type of exposure (e.g., anodal or cathodal stimulation). Anodal stimulation increases cortical excitability whereas cathodal decreases it.

As the SSM phenomenon has assumedly similar psychological mechanisms likewise the “attentional blink,” we can assume similar neurophysiological mechanisms (located in particular on the right PPC). In the current experiment, we investigated the involvement of the right PPC in the SSM phenomenon by using tDCS. We used a classical SSM paradigm (Fleck et al., 2010). In our adapted paradigm described below, each trial could include one high-salient or low-salient target, two targets (one high-salient and one low-salient target; two high-salient targets; two low-salient targets) or no target stimuli (catch-trials). Cathodal stimulation was applied over the right (target site) or left (control site) PPC. Sham stimulation over the right PPC was included as a control (placebo). We expected cathodal tDCS on the right PPC to increase the magnitude of the SSM (the difference in accuracy for single and dual target conditions) compared to stimulation of left PPC and to sham. In dual-target trials, targets of different saliency level (2 high-salient, 2 low-salient, and 1 high-salient + 1 low-salient) were used to manipulate the similarity. Based on the perceptual set theory, we expected the magnitude of SSM effect to be increased for the 1 high-salient + 1 low-salient target condition as compared to 2 high-salient and 2 low-salient target conditions. Additionally, we expected tDCS to modulate the SSM effect in 1 high-salient + 1 low-salient target condition.

## Materials and Methods

### Participants

Twenty-nine students of National Research University Higher School of Economics were enrolled into the study. Data of 5 participants were excluded due to high error rate (less than 30% of correct responses, presumably because these subjects did not take the experimental task with responsibility). The final sample included results of 24 participants (22 female and 2 male, from 18 to 21 years old, *M* = 19.45, *SD* = 0.8). All of them were native Russian speakers with normal or corrected to normal vision and unaware of the experiment purpose. All participants signed an informed consent before starting the experiment. The study was performed according to the Declaration of Helsinki and approved by the local Ethics Committee of HSE University.

### Stimuli and Apparatus

T-shaped “target” stimuli were presented among L-shaped distractors on a gray background (CIE xy = 0.273, 0.304; luminance = 40.897 cd/m^2^). Stimuli size was set at 1.76° × 1.76°. The stimuli color was gray with three different levels of salience – contrast respect to the background (CIE xy = 0.272, 0.297; luminance = 14.155 cd/m^2^ or CIE xy = 0.272, 0.301; luminance = 21.653 cd/m^2^ or CIE xy = 0.272, 0.303; luminance = 28.475 cd/m^2^). There were the same proportions of low/medium/high salience distractors on the display for each trial. The background color had the following intensities along the RGB channels: 128, 128, 128. L-shaped distractors had the following intensities along the RGB channels: 70, 70, 70; 90, 90, 90; and 105, 105, 105. T-shaped “target” stimuli had the following intensities along the RGB channels: 70, 70, 70 (high-salient target) and 105, 105, 105 (low-salient target). There were always 20 items presented for each trial. On each trial, one, two or no targets could be present. In the case of two targets presentation, they could have identical or different level of salience – contrast respect to the background (both high-salient, both low-salient or one high-salient and one low-salient). The positions of the stimuli were randomized. There were “OK” (6.85° × 4.32°) and “NO” (6.85° × 4.32°) rectangles at the bottom of the screen. Participants used these rectangles for response by using a computer mouse button.

Participants sat on comfortable chairs in a dark room in front of a 19 inches LACIE electron 19 blue III monitor (screen resolution 1024 × 768, refresh rate 85 Hz). The distance from the screen was 47 cm. Stimuli presentation was run by PsychoPy v. 1.82.01 software via OS Ubuntu. Participants’ responses were registered with a standard mouse.

### tDCS

Transcranial direct current stimulation was delivered by a battery driven, constant current stimulator (BrainSTIM by EMS, Italy) using a pair of surface saline-soaked sponge electrodes. A 5 cm × 7 cm electrode was used both for the site of stimulation and for the reference. The international 10–20 system for EEG electrode placement (Herwig et al., 2003) was used to apply a tDCS monopolar montage by placing the target electrode over the right (target site, over P4) or left (control site, over P3) PPC depending on the experimental condition, while the reference (anode) was placed over the ipsilateral shoulder (Cogiamanian et al., 2007; Feurra et al., 2010; Santarnecchi et al., 2014; Yaple et al., 2017). The current flow was applied for 10 min with at an intensity of 1500 μA (Ardolino et al., 2005). The current density at the stimulation electrode corresponded to 21.4 mA/cm^2^, below 25 mA/cm^2^, in order to avoid any adverse effect (McCreery et al., 1990). Sham (placebo) stimulation was delivered for 30 s at the beginning and at the end of stimulation, in order to induce subjects to feel an itching sensation that is usually felt during the rising up of real tDCS and it goes to diminish during the time course of the experiment. This short-lasting sham tDCS does not produce any after-effect (Nitsche and Paulus, 2000). For sham condition the target electrode was placed over the right PC which was the initially hypothesized target site. Stimulation was delivered offline.

### Procedure

The experiment was conducted in three sessions (according to different stimulation conditions) in three different days. A training session of 24 trials preceded the stimulation. After the stimulation was completed, participants had to perform the main session of the experiment. The sequence of presentation of stimulation conditions was counterbalanced across subjects (6 sequences of presentation for 3 conditions were made) and delivered in 3 different days, with at least a one-day break and not more than four-day break. Each session consisted of 300 trials. 50 trials were target-absent, 50 trials included one high-salient target, 50 trials included one low-salient target, 50 trials included two low-salient targets, 50 trials included two high-salient targets and 50 trials included one high-salient and one low-salient target. The order of presentation was randomized. The participant’s task was to detect all targets or to report their absence. Each trial started when the participant pressed “Space” bar on the keyboard. Each trial ended when the participant performed two mouse clicks (if no clicks were made, a next trial started after 20 s). In the case of no targets, participant had to point the mouse cursor on “NO” rectangle on the screen and to perform two mouse clicks. In the case of one target, the participant had to point the mouse cursor on the target and to perform a click and then to point the cursor on the “OK” rectangle and click. In the case of two targets, participant had to point the mouse cursor and to perform a click sequentially on each target.

**FIGURE 1:**
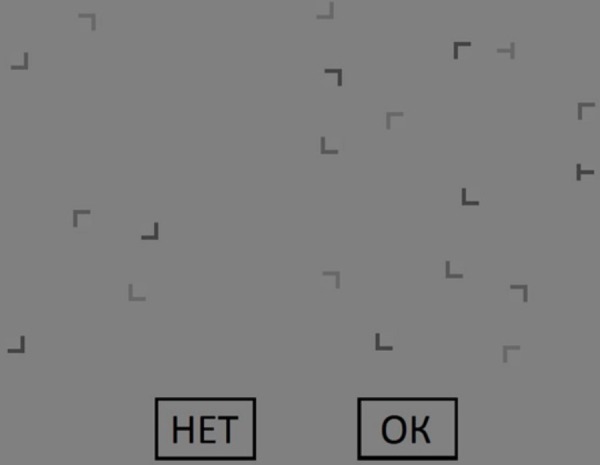
An example of the experimental trial. The target is the form of the letter “T.” There are two targets: one high-salient and one low-salient target. The button “HET” means “NO.”

The example of the trial design is shown in Figure 1.

### Data Analysis

Accuracy and reaction times (RTs) were computed. Accuracy was calculated with classical SSM measurement procedure. For dual-target trials, accuracy for the second target was calculated only for trials when first target was found correctly. For one high-salient and one low-salient dual-target trials, accuracy was calculated only for trials when high-salient target was firstly identified (the standard technique for calculating the SSM errors, e.g., Adamo et al., 2013). For two high-salient and two low-salient dual-target trials, accuracy was calculated only for trials in which at least one target was found [the technique for calculating the SSM errors for same-salience targets, e.g., (Gorbunova, 2017)]. RTs were analyzed separately for the first and second mouse clicks. RTs were analyzed only for correct response trials. RTs that deviated more than 2 SD’s from the mean were excluded from the analysis.

Data analysis was performed using SPSS 22.0. Repeated measures analyses of variance (rmANOVA) and pairwise comparisons were performed. The rmANOVA included the factor stimulation (stimulation of the right or left PPC and sham of the right PPC) and the factor type of target (one high-salient target; one low-salient target; two low-salient targets; two high-salient targets; one high-salient and one low-salient target; no targets). In the presence of significant interactions, corrected pairwise comparisons were performed by Bonferroni multiple comparisons test. The level of significance was set at *p* = 0.05. Greenhouse-Geisser corrections were applied when necessary to compensate for the violation of the assumption of sphericity.

## Results

### SSM Errors (T2|T1 Analysis)

**FIGURE 2:**
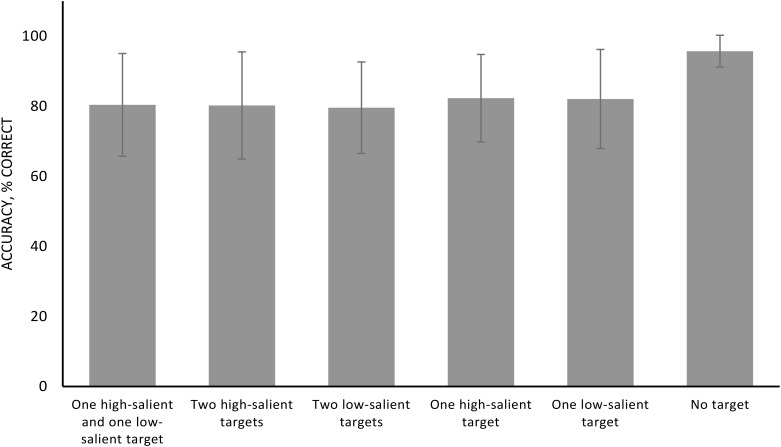
Accuracy (percentage correct) collapsing different stimulation conditions. Error bars represent standard error of the mean.

Results revealed a significant influence of the type of stimulation (*F* = 3.579, *p* = 0.050, *η^2^* = 0.135) and of the type of target (*F* = 22.615, *p* < 0.001, *η^2^* = 0.496). The interaction was not significant (*F* = 0.233, *p* = 0.969, *η^2^* = 0.010). The Bonferroni corrected pairwise comparisons of stimulation did not reveal any significant differences between tDCS on the RPPC and on LPPC, as between tDCS on the RPPC and sham on the RPPC, and between tDCS on the LPPC and sham on the RPPC (see Table 1 for detailed *p*-values). The Bonferroni corrected pairwise comparisons for type of target revealed significant differences between target absent and all other conditions (*p* < 0.001 for each). Other comparisons were not significant (all *p* > 0.05). The results are presented in Figure 2 and Tables 1, 2.

**Table 1:** Means and standard deviations (SD) in accuracy for different stimulation conditions.

	tDCS on the right PPC		tDCS on the left PPC		Sham on the right PPC	
	Mean	*SD*	Mean	*SD*	Mean	*SD*
One high-salient and one low-salient target	82.29	15.67	77.92	15.71	80.93	14.68
Two high-salient targets	83.29	13.75	77.51	19.84	79.84	15.28
Two low-salient targets	81.67	14.34	77.17	15.67	79.91	13.05
One high-salient target	83.75	15.57	80.08	14.77	83.08	12.47
One low-salient target	84.33	12.95	80.33	15.39	81.50	14.15
No target	97.41	2.85	93.92	7.04	95.83	4.53


**Table 2:** Pairwise comparisons (Bonferroni corrected) between different types of stimulation (RPPC, the right posterior parietal cortex; LPPC, the left posterior parietal cortex).

(I)Stimulation	(J)Stimulation	*P*-value, Bonferroni corrected
tDCS on the RPPC	tDCS on the LPPC	0.124
	Sham on the RPPC	0.397
tDCS on the LPPC	tDCS on the RPPC	0.124
	Sham on the RPPC	0.393
Sham on the RPPC	tDCS on the RPPC	0.397
	tDCS on the LPPC	0.393


### Reaction Time

**FIGURE 3:**
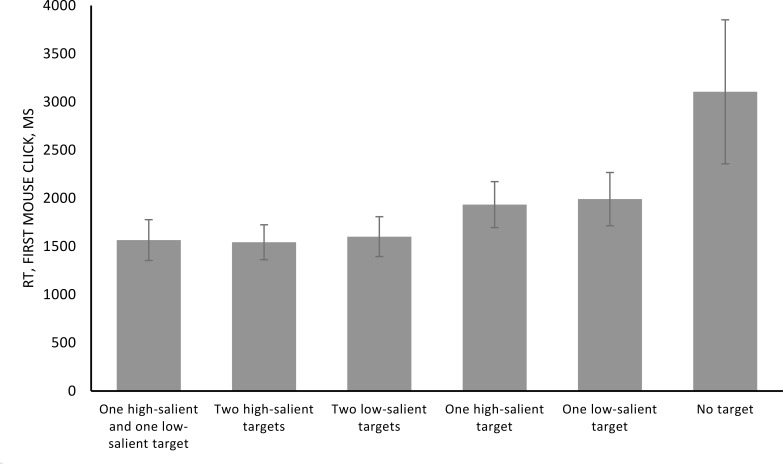
Mean reaction time (ms) of the first mouse click collapsing different stimulation conditions. Error bars represent standard error of the mean.

The rmANOVA (Greenhouse-Geisser corrected) revealed significant influence of the factor of type of target for the first mouse click time (*F* = 177.567, *p* < 0.001, *η^2^* = 0.885). The stimulation and the between-factors interaction were not significant (*F* = 0.58, *p* = 0.940, *η^2^* = 0.003; *F* = 0.441, *p* = 0.740, *η^2^* = 0.019). The pairwise comparisons revealed significant differences between conditions with one target and two targets (*p* < 0.001) and between the target absent and all other conditions (*p* < 0.001 for each). Other comparisons are not significant (all *p* > 0.05). The results are presented in Figure 3 and Table 3.

**Table 3:** Means and standard deviations (SD) in reaction time (ms) of the first mouse click for different stimulation conditions.

	tDCS on the right PPC		tDCS on the left PPC		Sham on the right PPC	
	Mean	*SD*	Mean	*SD*	Mean	*SD*
One high-salient and one low-salient target	1558.36	232.31	1559.94	281.82	1580.12	211.68
Two high-salient targets	1567.19	221.10	1552.56	260.84	1510.83	181.00
Two low-salient targets	1582.19	214.38	1587.27	242.77	1634.73	206.65
One high-salient target	1911.92	316.61	1927.02	349.08	1962.99	239.13
One low-salient target	1962.07	310.20	1995.96	378.31	2016.39	276.27
No target	3112.16	764.18	3078.17	558.63	3123.45	746.61


**FIGURE 4:**
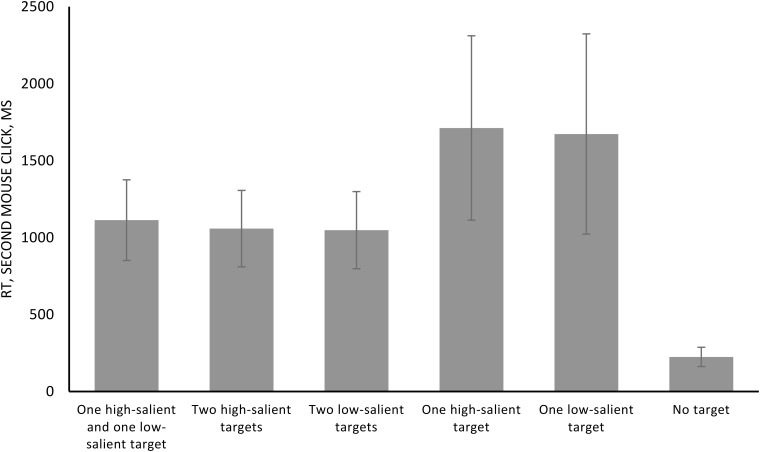
Mean reaction time (ms) of the second mouse click collapsing different stimulation conditions. Error bars represent standard error of the mean.

The results of the current study revealed a significant influence of the factor of the number of targets for the second mouse click time (*F* = 101.103, *p* < 0.001, *η^2^* = 0.815). The type of stimulation (*F* = 0.203, *p* = 0.780, *η^2^* = 0.009) and the interaction were not significant (*F* = 0.731, *p* = 0.552, *η^2^* = 0.031). The pairwise comparisons revealed significant differences between conditions with one target and two targets (*p* < 0.001 for each), between conditions with one high-salient, one low-salient target and two low-salient targets (*p* = 0.019), and also between the target absent and all other conditions (*p* < 0.001 for each). Other comparisons are not significant (all *p* > 0.05). The results are presented in Figure 4 and Table 4.

**Table 4:** Means and standard deviations (SD) in reaction time (ms) of the second mouse click for different stimulation conditions.

	tDCS on the right PPC		tDCS on the left PPC		Sham on the right PPC	
	Mean	*SD*	Mean	*SD*	Mean	*SD*
One high-salient and one low-salient target	1097.94	156.04	1106.15	260.69	1136.53	261.97
Two high-salient targets	1056.50	168.09	1034.09	211.57	1084.36	248.03
Two low-salient targets	1023.39	163.18	1052.78	249.27	1069.27	250.58
One high-salient target	1756.84	653.43	1670.80	584.55	1709.03	599.12
One low-salient target	1711.21	668.83	1636.93	628.69	1670.83	650.31
No target	238.09	102.69	218.46	47.16	217.53	62.66


## Discussion

Whereas the general effect of stimulation factor on visual search accuracy was found (*p* = 0.050), pairwise comparisons revealed no significant differences between any of the stimulation conditions, and no interaction was revealed, thus the suppression of right posterior parietal area did not lead to significant changes in the visual search accuracy. There are several explanations for these results.

One might be that the phenomenon of SSM is really not related to the right PPC. In that case, the question of localization of this phenomenon and its relation to attention remains open. According to the resource-depletion theory, cognitive resources (e.g., attention and/or working memory) are spent on finding the first target, and not enough resources are left for the second target search. For example, the subject captures the location or identity of the first target, thereby consuming the resources of attention and/or working memory. At this end, cathodal stimulation of right PPC was supposed to decrease the amount of available resources, and increasing the magnitude of SSM effect. Indeed resource depletion may still occur, but no attentional resources fall under depletion as expected. Still, WM resources are involved.

The second point is the insufficient suppressive power of tDCS for significant changes. In some studies, the lack of significant effects when using tDCS was pointed out. In the experiment of London and Slagter (2015), the obtained data indicated that the effect of tDCS was observed only during (but not after) anodal stimulation, whereas no effects were observed at the group level which emphasized the importance of taking into account the initial individual differences (individual excitation – inhibition balances might vary across and determine the effect of brain stimulation). Wang et al. (2018) conducted an experiment in which subjects performed a digit span tests and a visual short-term memory task before and after anodal tDCS. The results showed that stimulation of the left dorsolateral prefrontal cortex (DLPFC) does not affect the intentional digit span memory performance regardless of the time of the task. In addition, stimulation administered before the task did not affect visual short-term memory while there was a tendency to increase false alarm when stimulating the DLPFC during the task. Also, the possible limitation could be that the stimulation took 10 min, although more reliable protocol assumes 15 min of stimulation (e.g., Woods et al., 2016), and offline protocol. The question about offline and online stimulation is still controversial in literature (e.g., Das et al., 2016; Woods et al., 2016). However, many studies on WM, perception and attention used offline protocol reliably to get significant results (e.g., Ohn et al., 2008; Heinrichs-Graham et al., 2017). tDCS induces offline detectable LTP-like phenomenon (e.g., Amadi et al., 2015) that is why we were expecting such an effect while technical limitations (EEG/online combined tDCS) make it very difficult to investigate online mechanisms. The question about the time of stimulation (and intensity) is also controversial in literature. There are many studies that adopt different manipulation of parameters of stimulation (e.g., intensity and duration) and of size of stimulation electrodes [see (Fertonani and Miniussi, 2017)]. Here, we used 10 min intensity in order to be safe in terms of neurosensory side effects to the subjects (Fertonani et al., 2015; Fertonani and Miniussi, 2017). In any case, this might be a possible limitation of our study, and further study with online protocol or longer stimulation time might be a good control for our data.

Moreover, we did not find significant differences in response accuracy between single and dual target conditions, indicating the absence of an SSM. This can also be explained by the relative ease of the task. More complex stimuli are usually used in studies of SSM errors, whereas our experiment included simple T and L letters. In addition, some previous studies used a special gray “cloud” background for stimuli presentation (e.g., Fleck et al., 2010), which assumes more complexity as compared to our experiment.

Nevertheless, RT analysis revealed a significant difference between conditions with one and two targets. In single-target condition, the time of the first mouse click was longer than in dual-target condition. In dual-target trials, the time required to find at least one of two targets was less than the time required to find a single target in single-target trials. This result may depend on the fact that in a condition with two targets, the probability of finding a target is higher than in a condition with a single target when scanning the visual field. In addition, the RTs of the second mouse click for a condition with a single target (when the second click is the “OK” rectangle) is increased as compared to a condition with two targets. Similar results were obtained in the standard visual search task with one target. The RT is increased in trials without a target compared to those ones when the target is present (Kwak et al., 1991). In our experiments, the second mouse click in single-target condition assumed the target-absent report. These results are also similar to those obtained in previous study (Gorbunova, 2017).

A difference between conditions with two different targets (one high-salient, one low-salient target) and two low-salient targets was revealed. The RT was higher in the condition with two different targets. Such results may suggest the role of perceptual set. After finding the low-salient target, the subject is biased to find another low-salient target, so it takes longer to switch to high-salient target search. However, no difference was observed between two high-salient targets condition and two different targets condition, which may be related to additional modulation of perceptual set by salience factor.

It is worth noting that the higher accuracy in the subjects’ responses in the target-absent condition correlates directly with the theory of depletion of cognitive resources that are spent on finding the first targeted stimulus, thereby consuming attention resources and/or working memory, leaving fewer resources available for subsequent searches. Therefore, in the target-absent condition, resource consumption is minimal.

Overall, it can be concluded that we did not reveal the influence of tDCS stimulation over the right PPC on visual search accuracy and RTs performance. This may be associated with the lack of impact of this area to the chosen task or to the insufficient suppressive power of tDCS protocol design for inducing significant changes.

## Author Contributions

AL contributed to code, data collection, data analysis, and article preparation. MF contributed to general idea, experiments planning, and article preparation. EG contributed to general idea, experiments planning, code, and article preparation.

## Conflict of Interest Statement

The authors declare that the research was conducted in the absence of any commercial or financial relationships that could be construed as a potential conflict of interest.
